# Nutritional and Rheological Features of Lentil Protein Isolate for Yoghurt-Like Application

**DOI:** 10.3390/foods10081692

**Published:** 2021-07-22

**Authors:** Theresa Boeck, Emanuele Zannini, Aylin W. Sahin, Juergen Bez, Elke K. Arendt

**Affiliations:** 1 School of Food and Nutritional Sciences, University College Cork, T12 YN60 Cork, Ireland; theresa.boeck@umail.ucc.ie (T.B.); e.zannini@ucc.ie (E.Z.); aylin.sahin@ucc.ie (A.W.S.); 2 Fraunhofer Institute for Process Engineering and Packaging, Giggenhauser Str. 35, 85354 Freising, Germany; juergen.bez@ivv.fraunhofer.de; 3 APC Microbiome Ireland, University College Cork, T12 YT20 Cork, Ireland

**Keywords:** fermentation, pulses, lactic acid bacteria, plant-based

## Abstract

The substitution of animal protein with proteins of plant origin is a viable way to decrease the negative impact caused by animal husbandry on the environment. Pulse consumption has been widely promoted as a nutritious contribution to protein supplementation. In this study, an emulsion of lentil (*Lens culinaris*) protein isolate is fermented with lactic acid bacteria (LAB) to manufacture a yoghurt alternative and the techno-functional properties compared to a dairy- and a soy-based product with similar protein contents. The yoghurt-like products are subjected to large and small deformation analysis, quantification of fermentable oligosaccharides, disaccharides, monosaccharides and polyols (FODMAP), water holding capacity tests, protein profile analysis and the gel structure is visualised by confocal laser scanning microscopy (CLSM). The lentil yoghurt alternative shows good water holding capacity, high firmness and consistency values in large deformation analysis, with cohesiveness and viscosity not significantly different from that of dairy yoghurt. The high gel strength and rigidity of the lentil yoghurt gels measured by small deformation analysis is well-reflected in the dense protein matrix in the CLSM graphs. FODMAP content of the lentil yoghurt is very low, making it suitable for consumption by irritable bowel syndrome (IBS) patients. Our results show that lentil protein isolate is an excellent base material for producing a plant-based yoghurt alternative.

## 1. Introduction

The growth of the world population and the strains put on crops by climate change will pose a challenge to global food security in the years to come [1]. Due to the high amount of protein and nutrients in pulses, increasing global pulse consumption could significantly contribute to both food and protein security [2]. Partially substituting dairy products with pulse-based products also significantly decreases greenhouse gas emissions [3], the usage of land and water [4], as well as nitrogen fertiliser [5]. A diet rich in pulses has also been shown to have considerable health benefits, such as lower risks of diabetes, coronary heart disease or stroke [6,7,8,9].

Currently, pulses are mainly grown in developing countries in the middle latitudes, where they play an essential part in food security and soil health, due to their nitrogen-fixing ability [10]. While pulses currently only constitute 5% of the global daily protein intake, this number is much higher in some developing countries, making up >30% in Rwanda and >10% in India and Brazil [11]. Rising temperatures and more frequent droughts, due to climate change, threaten pulse yields in marginal environments. They also facilitate pulse production in areas previously too cold, such as northern areas of Europe or Russia [12,13]. 

The first legume widely consumed as a dairy substitute in the Western world was the soybean (*Glycine max*). In Asia, soy was traditionally used as a base for a drink similar to dairy milk and tofu for approx. two thousand years [14]. In recent times, there have been growing consumer demands for dairy alternatives for lifestyle and health reasons, as well as ethical and environmental concerns [15]. Between 2018 and 2020, the European market for plant-based yoghurt has increased by 37% to €405 million [16,17]. As the market for plant-based alternatives has grown, dairy alternatives made from grains and nuts have joined the soy products on supermarket shelves. The majority of plant-based yoghurt alternatives currently on the market use coconut, soy or almond as base ingredients. However, many of these products lack the protein content provided by dairy and soy products, and therefore, do not meet the criteria to be labelled as sources of protein in the EU (>12% of the energy value of the product is provided by protein) [18,19,20,21]. Adequate dietary protein uptake is essential for maintaining health, and as plant-based yoghurt alternatives are often used to replace dairy-based products in the diet, it is crucial that these substitutes provide the consumer with similar protein contents [22]. While soy-based yoghurt alternatives typically are high in protein [23], soy is a common allergen [24,25]. Previous studies have successfully investigated the suitability of other pulses and pulse protein isolates as the base material for yoghurt alternatives, such as lupin protein isolate [26], black beans [27] or faba bean flour [28]. It has been shown that lentil protein isolate can be used to produce a milk alternative with a protein content equal to that of dairy and soy products and good sensory and techno-functional properties [29]. However, to the authors’ knowledge, no studies investigating lentil protein as the base material for fermented dairy alternatives have been published. In this paper, lentil protein isolate has been tested for its suitability as a base material for a novel alternative to dairy yoghurt. Fermentation of plant milk can increase both the palatability, as well as the nutritional value of plant milks [30,31]. Fermenting with standard strains of *Streptococcus thermophilus* and *Lactobacillus bulgaricus* tends to show high levels of post-acidification during cold storage after fermentation, altering the rheological properties of protein gels [32]. Therefore, samples for this study were inoculated with a mixture of lactose-negative, sucrose-fermenting strains of *S. thermophilus* and *L. bulgaricus*, which ensures a comparable pH between samples and pH stability after the end of fermentation. Thus, the techno-functional behaviour of a lentil protein isolate emulsion upon acidification by lactic acid bacteria fermentation was studied and compared to fermented cow’s milk and soy drink. A lentil-based yoghurt-alternative could pose a viable way towards increasing pulse consumption in consumers in Western countries and provide a nutritious, low carbon footprint alternative to both dairy and soy-based yoghurt products. 

## 2. Materials and Methods

### 2.1. Materials

Lentil protein isolate (LPI) was produced by aqueous extraction and isoelectric precipitation of brown lentils and was provided by Fraunhofer Institute for Process Engineering and Packaging, Freising, Germany. A detailed characterisation of the LPI has been previously published by Alonso-Miravalles et al. [33]. As controls, semi-skimmed cow’s milk (Tesco Low Fat Milk), and a commercial soy drink (Rude Health Soya Drink Organic) chosen because of its protein and fat content similar to cow’s milk, were used. For inoculation, Yoflex^®^ Acidifix^TM^ (Chr. Hansen, Hørsholm, Denmark) was used. A concentration of 0.75% sucrose in cow’s milk leads to acidification to pH 4.6. At this pH, all the sucrose has been metabolised, and no further acidification can occur, as these strains cannot ferment any present lactose. Chemicals were purchased from Sigma-Aldrich (St Louis, MO, USA) unless otherwise stated.

### 2.2. Preparation of Set Yoghurts

A lentil protein emulsion was produced using a modified protocol by Jeske et al. [29]. Briefly, 4.35% (*w*/*w*) LPI and 0.75% (*w*/*w*) sucrose were added to water and the LPI hydrated for an hour in a water bath at 50 °C, with brief mixing on a magnetic stirrer every 15 min. The solution was then sheared with an Ultra-Turrax T18 equipped with an S18N-19G dispersing element (IKA Labortechnik, Janke and Kunkel GmbH, Staufen im Breisgau, Germany) for 15 min at 6,000 rpm while simultaneously stirring on a magnetic stirrer. Sunflower oil was added to a concentration of 1.5% (*w*/*w*), and the solution was sheared again for 15 min. Due to the protein content of the LPI of 80.48% (*w*/*w*), the final lentil emulsion contained 3.5% (*w*/*w*) net protein. The solution was then homogenised in two passes on a two-stage high-pressure homogeniser (APV-2000, SPX FLOW Inc., Charlotte, NC, USA) at 800 bar (1st stage 100 bar). 

To ensure equal sucrose concentrations in the unfermented lentil emulsion, soy drink and dairy milk, the natural sucrose content of the lentil emulsion and the controls was measured by HPLC as described in Section 2.10 (data not shown) and the necessary amount of sucrose to reach a final concentration of 0.75% (*w*/*w*) was added. For pasteurisation and partial heat denaturation of proteins, the lentil emulsion and the dairy milk and soy drink control were heated to 85 °C for 2 min in a stirring water bath (Lochner mashing device LP electronic, Berching, Germany) [29]. For the fermentation, the lentil emulsion and the controls were cooled to 42 °C, inoculated with 0.03% (*w*/*w*) Yoflex^®^ Acidifix^TM^, stirred for 15 min in a water bath at 42 °C to ensure even distribution of the cultures, and incubated in sterile plastic containers and centrifuge tubes at 42 °C. After fermentation, samples were stored at 6 °C for 12 to 16 h prior to further analyses.

### 2.3. Fermentation Monitoring by pH and Total Titratable Acidity (TTA) Measurement

For pH and TTA measurements during fermentation, a part of the samples was incubated in 50 mL tubes, and the contents of one tube were measured every hour of fermentation and after 12 h of storage. pH was measured with a digital pH meter (SevenGo Mettler-Toledo Ag, Greifensee, Switzerland). TTA was determined using a modified version by Zannini et al. [34]. The contents of the tubes were homogenised, 15 g of sample suspended in 35 g of water and titrated with 0.1 M NaOH to a pH of 8.5. After 3 min, pH was readjusted to 8.5 if necessary. TTA was expressed as mL NaOH per g of sample. Fermentation was ended if a pH of 4.5 was reached, or the pH did not drop further in 2 consecutive measurements 1 h apart. 

### 2.4. Viable Cell Count

Viable cell count after inoculation and at the end of fermentation was determined by preparing serial dilutions in sterile Ringer’s solution and plating on deMan, Rogosa and Sharpe (MRS) agar supplemented with 0.05 g/L bromocresol green and 2% sucrose. Plates were incubated anaerobically at 37 °C for 72 h before colony enumeration. 

### 2.5. Water Holding Capacity

The amount of liquid expulsion was determined by centrifugation and drainage method, two different methods commonly used in traditional yoghurt analysis [35]. For the centrifugation method, adapted from Brückner-Gühmann et al. [36], samples of a defined weight (m_start_) fermented in 50 mL tubes were centrifuged at 200× *g* for 10 min at 6 °C. The supernatant was then removed with a pipette, and the samples were weighed again (m_end_). The liquid expulsion was calculated using Formula (1):

LE (%) = ((m_start_ − m_end_)/m_start_) × 100 (1)

For the drainage method, adapted from Harwlakar et al. [37], the content of individual sterile cups (m_start_) was transferred onto a funnel lined with a paper filter (Whatman^®^ qualitative filter paper, grade 113), taking care not to unnecessarily disrupt the sample structure. The samples were then left to drain at 6 °C for 2 h, with the expulsed liquid being collected in a beaker (m_liquid_). The liquid expulsion was calculated using Formula (2):

LE (%) = (m_liquid_/m_start_) × 100 (2)

### 2.6. Uniaxial Compression Testing

Textural properties of the fermented samples were measured by a back-extrusion method using a TA-XT2i Texture Analyser (Stable Micro Systems, Surrey, UK) equipped with a 25 kg load cell and an extrusion disc (Ø = 35 mm), according to a method by the authors of [38] with slight modifications. Two hundred millilitres of milk was fermented in individual sterile cups, so the structure did not have to be disrupted prior to measurement. Measurements were performed immediately after removal from storage (24–30 h at 6 °C). The test was performed at a probe speed of 1 mm/sec, a penetration depth of 20 mm and a trigger force of 0.05 N, and the force-time curves were evaluated using the Exponent software (Stable Micro Systems Ltd., Godalming, Surrey, UK). The measured factors were firmness (maximum positive force), consistency (positive area of the curve), cohesiveness (maximum negative force of the curve) and viscosity index (negative area of the curve) [18].

### 2.7. Rheological Measurements

Small deformation rheological measurements were carried out using a controlled stress rheometer (MCR301, Anton Paar GmbH, Graz, Austria) equipped with a concentric cylinder measuring system (C-CC27-T200/SS, Anton Paar GmbH, Austria). All measurements of the fermented samples were performed at a sample and rheometer temperature of 6 °C. The samples were stirred 10 times with a spatula before transferring them to the rheometer cup and then left to equilibrate in the rheometer for 15 min prior to the measurements. 

Frequency sweeps were performed at frequencies from 0.01–15 Hz at a constant strain of 0.1%. For the evaluation of gel behaviour, the slopes of G′, G″ and |η*| vs. the angular frequency ω = 2πf in a log-log graph were calculated.

The three interval thixotropy test was performed using a method adapted from [39]. The first, oscillatory, interval was performed at a strain of 0.1% and a frequency of 1 Hz for 120 s, followed by an interval of rotational deformation at a shear rate of 200 s-1 for 120 s. The third interval was performed at the same conditions as the first interval, but lasted for 300 s. Structure loss by this intense shearing was calculated as follows:

ΔG′ [%] = 100% − (G′_end_ × 100%)/G′_start_) (3)

ΔG″ [%] = 100% − (G″_end_ × 100%)/G″_start_) (4)

The structure formation during the fermentation of the milks was evaluated by fermentation in the rheometer. This facilitated an analysis of the samples without disruption of the structure. After inoculation, the samples were added to the cup of the rheometer, covered with a thin layer of vegetable oil to avoid evaporation, and fermented for 6 h at 42 °C during oscillatory analysis at a frequency of 1 Hz and a strain of 0.1%.

### 2.8. Confocal Laser Scanning Microscopy

Sample microscopy for protein structure analysis was performed on a confocal laser scanning microscope (CLSM) (Fluoview FV1000, with an IX81 inverted microscope; Olympus, Hamburg, Germany) on 100× oil immersion objectives) and a He–Ne laser (excitation wavelength 633 nm, emission detected between 565–615 nm).To facilitate the analysis of an undisturbed gel structure, samples were fermented in 8-well tissue culture chambers (Sarstedt, Hemer, Germany) [40]. After inoculation, samples were mixed with an aqueous 0.001% (*w*/*v*) solution of Nile blue to a concentration of 5% (*v*/*v*) of dye per sample. Samples were then fermented for 4 h (dairy control) or 5 h (lentil sample and soy control) and stored for 16 h at 6 °C prior to analysis. Unfermented samples were mixed with a saturated Nile blue solution to a final concentration of 33% (*v*/*v*) for analysis [29]. 

### 2.9. Colour Measurements

The colour of the non-fermented and fermented samples was measured with a Chroma Meter (Minolta CR-300, Osaka, Japan) using the CIE L*a*b colour space. The colour is mapped on Cartesian coordinates, with the L axis representing the lightness or luminosity, the a*-axis the red (positive values) or green (negative values) proportion of the colour, and the b*-axis the yellow (positive values) or blue (negative values) proportion. The whiteness index of the samples is calculated using Equation (5) [20]:

WI = 100 − √((100 − L*)^2^ + a*^2^ + b*^2^) (5)

### 2.10. Quantification of Sugars, Organic Acids and FODMAPs

Unfermented and fermented lentil samples and dairy and soy controls were diluted and filtered with a 0.2 μm polyamide syringe filter (Chromafil AO-20/25, Machery Nagel, Düren, Germany) before analysis. Sugars were quantified by HPLC on an Infinity 1260 system with a refractive index detector (Agilent Technologies, Palo Alto, CA, USA), using a Sugar-Pak I column (300 × 6.5 mm; Waters Corporation, Taunton, MA, USA), and an eluent of 0.0001 M CaEDTA at a flow rate of 0.5 mL/min and a column temperature of 80 °C. Maltotriose, sucrose, lactose, glucose, fructose, and mannitol were used as external standards. Organic acids were quantified on the same system, using a Hi-Plex H column (300 × 7.7 mm, 60 °C column temperature, Agilent) with a Hi-plex H guard column, 0.005 M H_2_SO_4_ as mobile phase at a flow rate of 0.5 mL/min. For quantification, citric, gluconic, malic, succinic, lactic, fumaric and acetic acid were used as external standards [34].

FODMAPs were quantified by high-performance anion-exchange chromatography coupled with pulsed amperometric detection (HPAEC-PAD), performed on a Dionex™ ICS-5000+ system (Sunnyvale, USA) as described in [41]. The relevant FODMAPs for pulse analysis were chosen according to [42]; additionally, lactose and sucrose content was quantified. Fructans are not present in pulses, and analysis was, therefore, omitted [42]. Lactose content was quantified using a Thermo Scientific Dione CarboPac PA200 column, analysis of all other FODMAPs and sucrose was performed on a Thermo Scientific Dionex CarboPac PA1 column. The data evaluation of both HPLC and HPAEC-PAD was performed with the software Chromeleon 7.2. 

### 2.11. Protein Profile Analysis

The molecular weights of the proteins in the fermented and unfermented lentil samples and dairy and soy controls, as well as in the soluble fractions after fermentation, was analysed using an Agilent Bioanalyzer 2100 Lab-on-a-Chip capillary electrophoresis system with an Agilent Protein 80 kit. For the soluble protein fraction, the fermented samples were centrifuged at 4000× *g* for 10 min, and the supernatant was used directly for the protein profile analysis. Proteins of the fermented and unfermented samples were extracted using the method from [43], using a protein concentration of 0.5% *w*/*v*. DTT was added for reducing conditions. As it has been shown that there are only minor differences between the reducing and non-reducing protein profiles of lentil protein using this method [33,44], non-reducing conditions have been omitted.

### 2.12. Statistical Data Analysis

All analyses were performed at least in triplicate. Results were analysed using a one-way analysis of variance (ANOVA). Means were compared by using Fisher’s least significant difference test (*p* < 0.05). All statistical analyses were conducted using Statgraphics Centurion XVI, Statpoint Technologies Inc., The Plains, VA, USA. 

## 3. Results

### 3.1. Fermentation Characteristics

**Figure 1:**
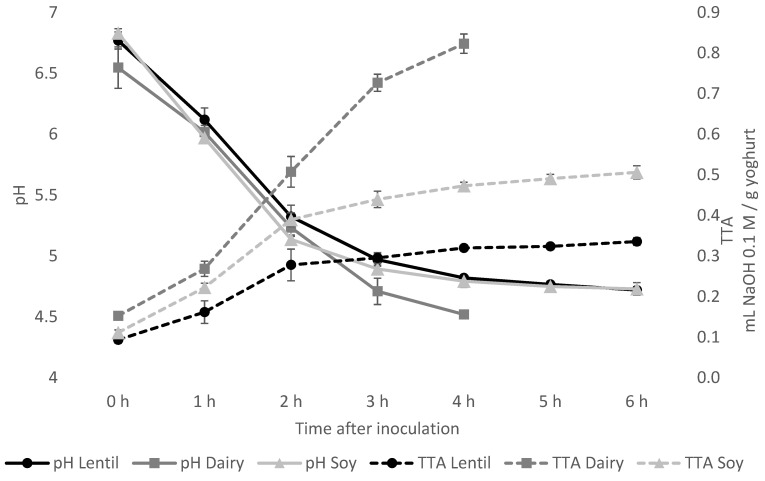
Acidification during fermentation as measured by pH (solid lines) and total titratable acidity (TTA) in ml NaOH 0.1 M per g of yoghurt (dashed lines). The fermentation was ended if a pH of 4.5 was reached, or the pH did not drop significantly in 2 consecutive measurements 1 h apart. Fermentation of dairy controls was ended after 4 h, so no data points for 5 h and 6 h are given for dairy controls.

The acidification kinetics were characterised by the drop in pH, as well as the increase in titratable acidity. As shown in Figure 1, the pH drop after inoculation was very similar in the lentil and soy fermentations. After 6 h of incubation, a pH of 4.72 ± 0.03 for the lentil sample and 4.73 ± 0.05 for the soy control was reached. The dairy control reached the desired pH of 4.52 ± 0.01 after 4 h of incubation. The lentil samples’ TTA value remained much lower than that of the soy and the dairy control, with 0.34 ± 0.01 mL 0.1 M NaOH/g sample for lentil, 0.51 ± 0.02 mL for soy and 0.82 ± 0.02 mL for the dairy control at the end of fermentation. 

These results are also reflected in the lactic acid content as measured by HPLC. Table 1 shows that the lactic acid content was much lower in the fermented lentil and soy samples than in dairy samples. As can be seen from the sucrose content in the fermented samples, this is due to an incomplete use of the sugar source in the lentil and soy samples. In the dairy samples, the sucrose was metabolised entirely at the end of fermentation, while in the lentil and soy samples, only 38% and 53% of the sucrose was fermented, respectively. The analysis of the viable cell count at the end of the fermentation, however, shows that there was no statistically significant difference between the cell counts of the lentil samples and the dairy and soy controls (Table 1). 

### 3.2. Water Holding Capacity

**Figure 2:**
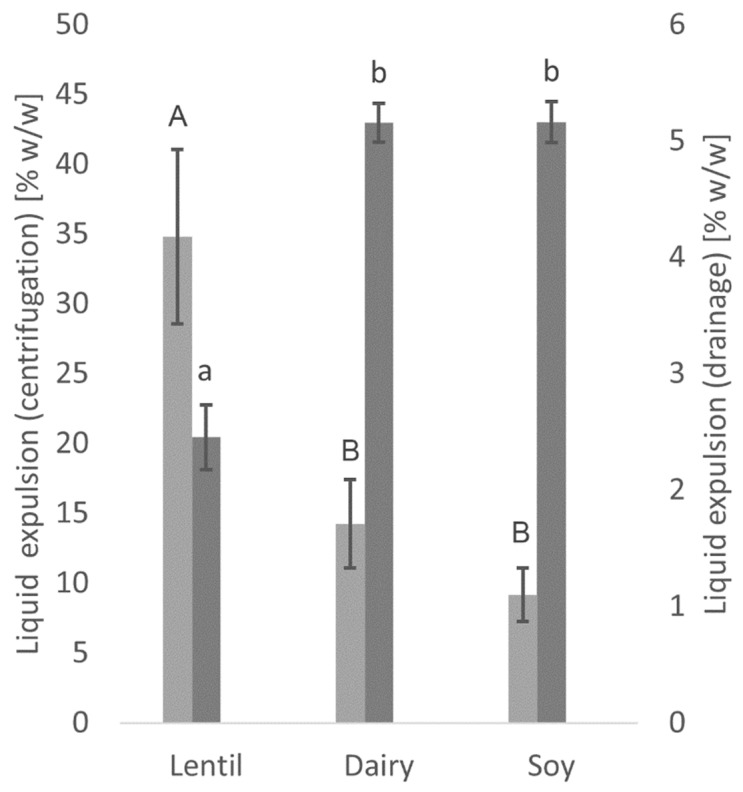
Liquid expulsion as measured with the centrifugation method (light grey) and drainage method (dark grey). Values with different superscript letters within columns for the centrifugation (capital letters) or drainage method (lowercase letters) are significantly different (*p* ≤ 0.05).

Significant differences can be seen in the water holding capacity between the lentil yoghurt and the dairy and soy controls, as well as between the centrifugation and the drainage method (Figure 2). While the lentil yoghurt showed more than twice the liquid expulsion when measured by centrifugation, only about half the amount of liquid was expulsed by drainage compared to the dairy and soy controls. No significant differences in liquid expulsion between dairy and soy controls were found with either method. 

### 3.3. Textural Properties

**Figure 3:**
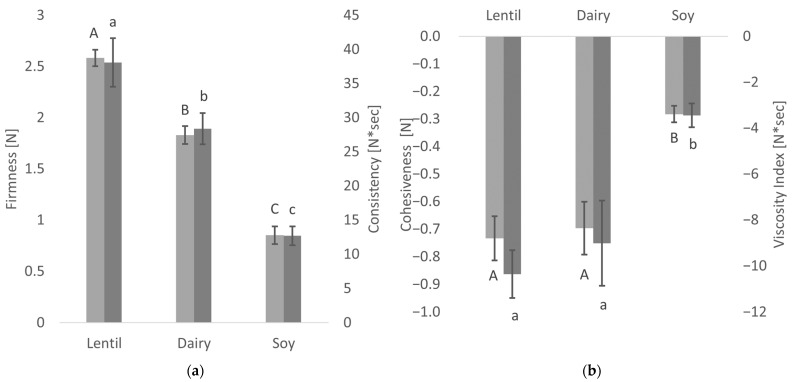
Textural properties of the fermented samples: Means of firmness (light grey) and consistency (dark grey) (**a**) and cohesiveness (light grey) and viscosity (dark grey) (**b**). Values with different superscript letters within columns for firmness and cohesiveness (capital letters) or consistency and viscosity index (lowercase letters) are significantly different (*p* ≤ 0.05).

The textural properties of the fermented samples as measured by large deformation analysis with the back-extrusion method are shown in Figure 3. These tests simulate the behaviour of the fermented samples when broken down during processing or in the mouth [26]. 

There were significant differences in firmness and consistency between the fermented lentil, dairy and soy samples. Lentil showed the highest values in firmness, as well as consistency, and was, therefore, firmer and thicker than the controls. In the soy control, both firmness and consistency were less than half of the lentil samples. The lentil sample and the dairy control show no significant differences in cohesiveness and viscosity index. However, the fermented soy control shows much lower values in both factors, indicating a more liquid, non-cohesive product. 

### 3.4. Gel Behaviour of Fermented Samples

**Figure 4:**
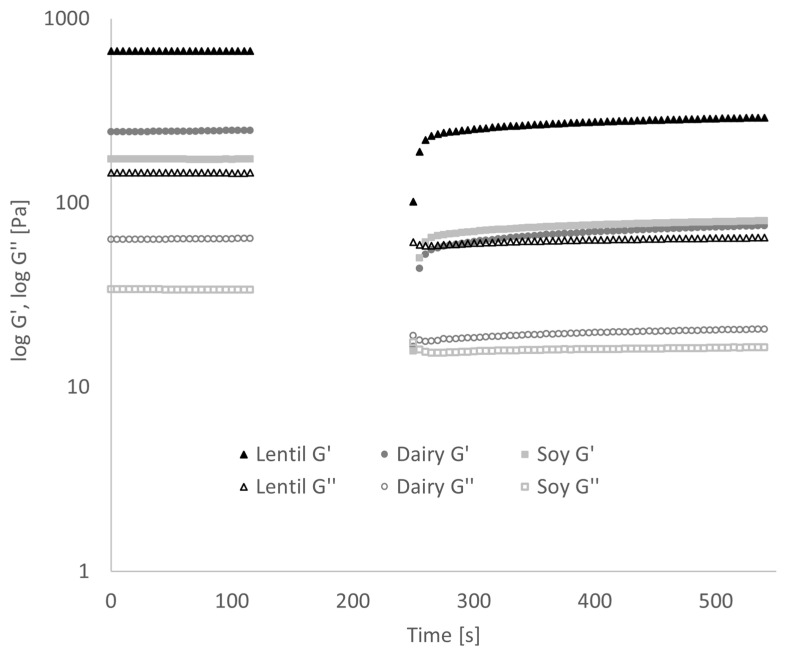
Results of the three interval thixotropy test. The gap in values between 120–240 s is due to test characteristics. Error bars have been omitted for clarity.

In the three interval thixotropy test, the first and the third interval are oscillation steps within the linear viscoelastic regime (established by amplitude sweeps, data not shown). In the second step, the gel structure of the fermented samples is ruptured by high shear rates. Over the third interval, the gel structure partially re-forms (see Figure 4). The difference in storage modulus G′, representing the elastic portion of the viscoelastic behaviour, and the loss modulus G″, which represents the viscous portion, between the end and beginning of the thixotropy test are shown in Table 2. In the dairy control, close to 70% of both G′ and G″ were lost after shearing. In the lentil and soy samples, losses only amounted to around 55%. 

**Figure 5:**
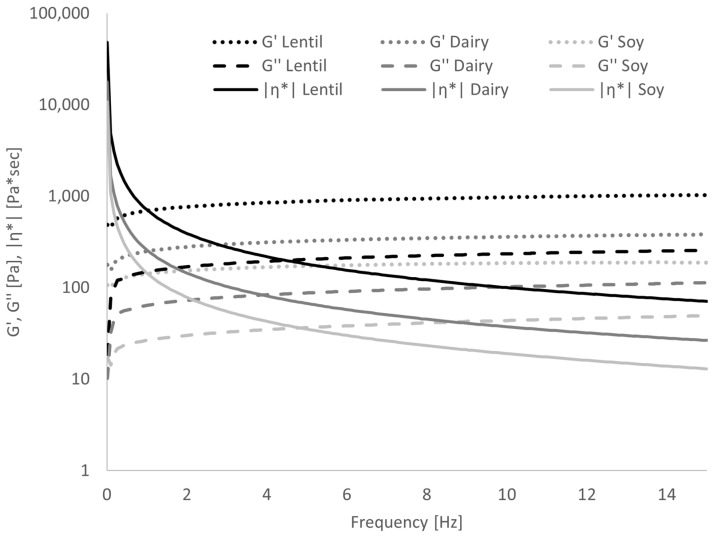
Results of frequency sweep: Dependence of storage modulus G′, loss modulus G″ and complex viscosity |η*| on frequency. Error bars have been omitted for clarity.

The frequency sweep (Figure 5) showed that in lentil samples, G′, and thus, the elastic or solid portion of the viscoelastic behaviour, was 4 to 4.5 times larger than G″ over the entire range of tested frequencies. In the dairy samples, G′ was approx. 3 to 4 times larger, and in the soy samples G′ was 4 to 5 times larger than G″. This corresponds to a damping factor tan(δ) (which is the ratio of G″ to G′) of 0.22–0.25 for lentil, 0.25–0.33 for dairy and 0.20–0.25 for soy samples. The values for tan(δ) can range from 0 to infinity, and the following ranges to categorise polymer systems has been proposed by [45]: Very high for low-concentrate solutions, 0.2–0.3 for amorphous polymer systems, low (close to 0.01) for gels. All three samples, therefore, present more as amorphous polymer systems than true gels, but the lentil and soy samples show significantly lower tan(δ) values, and thus, more gel-like behaviour than the dairy sample. In Table 2, the slopes of G′, G″ and the complex viscosity |η*| over the angular frequency in a log-log graph are given. In true gels, G′ and G″ are constant over frequency, while in solutions, they are a function of frequency [45]. As can be seen from the values of dlog G′/dlog ω and dlog G″/dlog ω, G′ and G″ are weakly positively correlated with frequency. No significant differences could be found between the lentil sample and the controls in either slope. The slope of dlog|η*|/dlogω in a true gel is −1 [39]; all samples differ strongly from this behaviour, with soy showing the lowest slope of complex viscosity.

**Figure 6:**
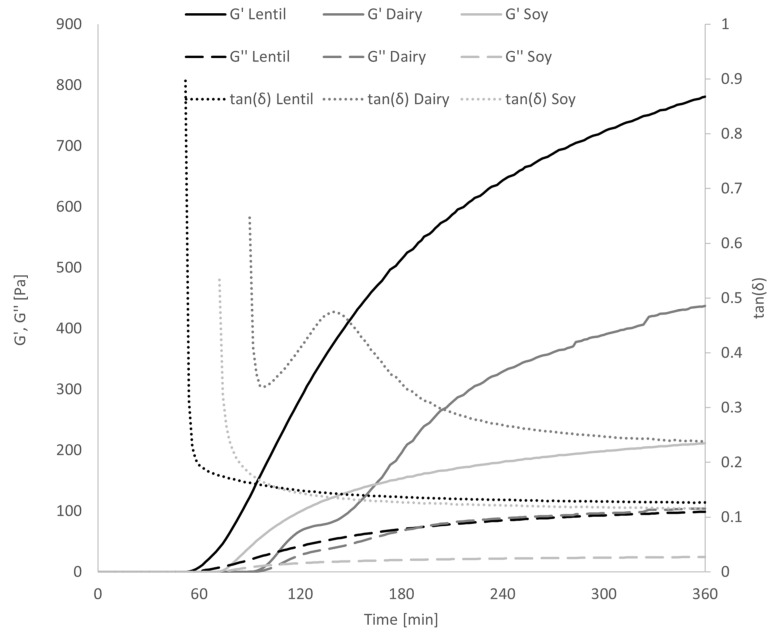
Development of a gel structure during fermentation in the rheometer, measured by G′ and G″ and tan(δ). Error bars have been omitted for clarity.

By fermenting the samples in the rheometer during oscillatory analysis, it could be shown that the gel formation progressed much faster in the lentil samples than the dairy or soy controls (Figure 6). At around 70 min fermentation time, G′ of the lentil sample grows exponentially while tan(δ) drops, indicating gel formation. Soy gel formation starts at around 90 min, while the dairy tan(δ) only decreases at 100 min. G′ of the dairy control grows exponentially between minutes 100 and 120 and plateaus for 20 min until exponential growth is reached again. 

### 3.5. Microscopic Protein Structure 

**Figure 7:**
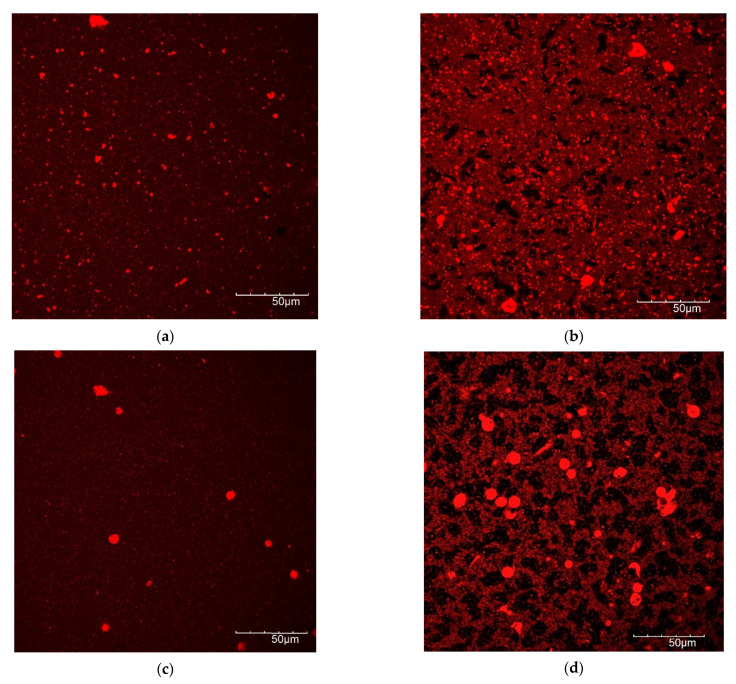

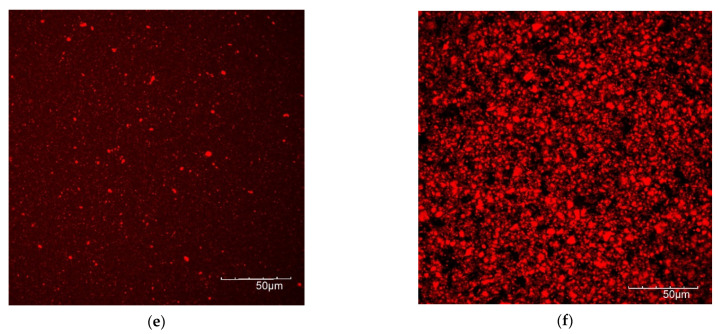
CLSM images of protein of non-fermented (left side) and fermented (right side) samples. (**a**,**b**) Lentil, (**c**,**d**) dairy, (**e**,**f**) soy.

In Figure 7, the protein structure before and after fermentation is shown by CLSM pictures, with the protein dyed with Nile blue. Fermentation of the lentil protein emulsion led to the formation of a very dense matrix with few, but large pores (Figure 7a,b). In contrast, the fermented dairy protein network formed a looser protein network than the lentil sample, with a higher number of pores, i.e., the black areas of the image (Figure 7c,d). The fermented soy control (Figure 7e,f) showed a weaker protein matrix with numerous, large pores. These results correspond well with rheological and textural measurements, where the fermented lentil samples proved to be firmer and more rigid gels than the dairy and soy controls. 

### 3.6. Colour Results

**Figure 8:**
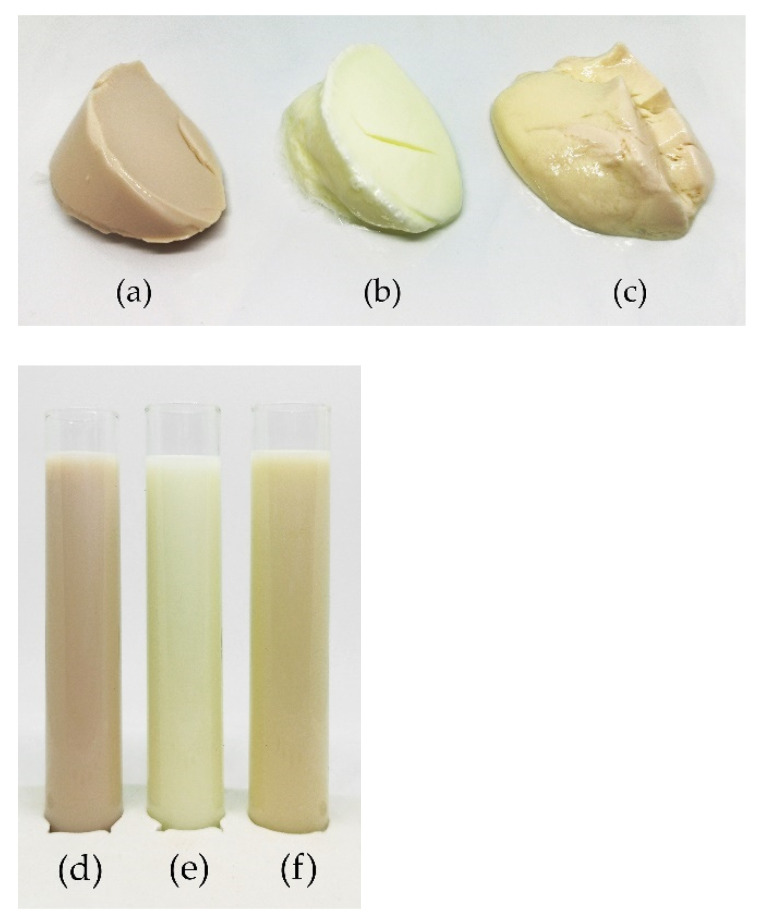
Photographs of the fermented samples, (**a**) lentil, (**b**) dairy, (**c**) soy and non-fermented milks, (**d**) lentil, (**e**) dairy, (**f**) soy.

As shown in Figure 8, the lentil samples are a pale pink colour, caused by carotenoids, anthocyanins, and flavonoids in the hulls of the brown lentils from which the lentil protein was isolated [46]. The dairy samples are light cream coloured, and the soy samples have a peachy yellow hue. The exact colour values using the CIE L*a*b colour space can be seen in Table 3. Fermentation increased the whiteness index values of both the lentil and the soy samples. The whiteness index value of the lentil samples rose from 80.14 to 85.63 after fermentation, which is close to the whiteness index value of the fermented dairy sample, 89.22 and slightly higher than that of the fermented soy sample.

### 3.7. FODMAP Content

A summary of FODMAP content is given in Table 4. The lactose content in the dairy samples only slightly decreased during fermentation, as the LAB contained in the Yoflex^®^ Acidifix^TM^ used for fermentation are lactose-negative. Glucose and fructose levels both increased significantly during fermentation in all samples. While none of the unfermented samples contained excessive fructose (EF), excess fructose, probably caused by acid hydrolysis of the sucrose, could be measured in lentil, dairy, and soy samples after fermentation. The amount of EF in the fermented lentil samples was only approximately half of the fermented dairy samples. The dairy samples did not contain polyols or galacto-oligosaccharides (GOS). Of polyols, only xylitol and sorbitol were present; mannitol could not be found in either plant-based sample. Fermentation decreased polyol content in the lentil samples from 30 to 4 mg/100 g, while in the soy sample, polyol content did not change significantly (13 to 11 mg/100 g). The GOS raffinose was only found in the soy sample, and the raffinose content was not changed significantly by fermentation. Stachyose content was much lower in the lentil than the soy product and was only slightly decreased by fermentation (62 to 53 mg/100 g in lentil, and 310 to 293 mg/100 g in soy).

### 3.8. Protein Profile

**Figure 9:**
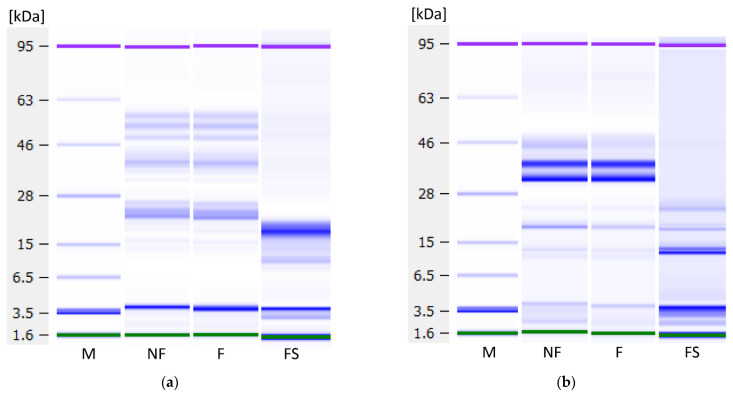

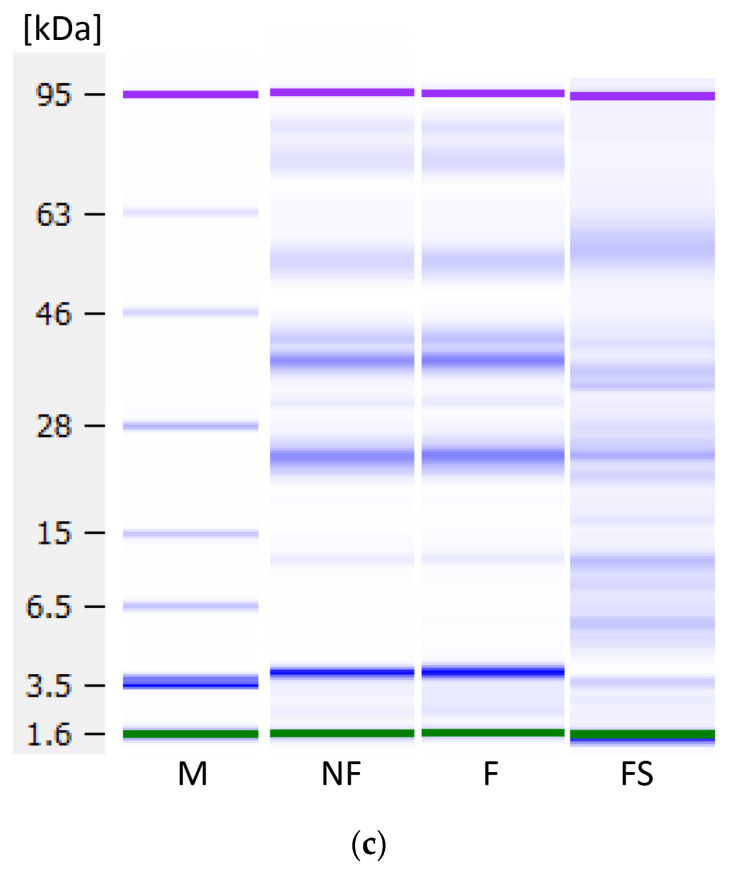
Protein profiles under reducing conditions as measured with the Agilent Bioanalyzer. (**a**) Lentil, (**b**) dairy and (**c**) soy samples. M—marker, NF—non-fermented samples, F—fermented samples, FS—fermented samples, soluble proteins. Green and purple bands are marker bands, and the bands at 3.5 kDa are system peaks that do not derive from the samples.

To investigate whether fermentation influenced protein profiles and analyse the proteins not precipitated by fermentation, Lab-on-a-Chip capillary electrophoresis was performed. Figure 9 shows the protein profiles under reducing conditions of the non-fermented and fermented samples and the soluble fractions’ profiles, i.e., the non-coagulated proteins present in the liquid fraction. No degradation of proteins has occurred during fermentation, indicating an absence of proteolytic activity by the used LAB strain. In the lentil samples (Figure 9a), three proteins around ~50 kDa, a protein at ~40 kDa and two proteins between ~26 and ~22 kDa could be observed, as well as weak bands at lower molecular weights. The 50 kDa protein may be one of the subunits of the 7S globulin vicilin. This trimeric protein of about 150 kDa is one of the major storage proteins in lentils. The subunits of vicilin are not connected by disulphide bridging, as the content of sulphur-containing amino acids is low [47,48]. The proteins at 40 and 26 to 22 kDa might correspond to subunits of the 11S globulin legumin, the second important lentil storage protein. Legumin consists of 6 polypeptide pairs of one acidic subunit (~40 kDa) and one basic subunit (~20 kDa) each, which are linked by one disulphide bond [49]. In the soluble fraction, a very strong band at a MW of ~19 kDa, and a weaker band at ~12 kDa were observed, both of which probably correspond to albumins. Lentil albumins have a pI of 6.00, and therefore, do not precipitate upon acidification, as opposed to globulins with a pI of 4.4–4.6 [50]. 

The dairy samples (Figure 9b) show α-lactalbumin at 14 kDa, β-lactoglobulin at 25 kDa, β-casein at 30 kDa, α_s_-casein at 32 kDa and κ-casein at 45 kDa, which follows previous analyses of bovine milk samples on the Bioanalyzer [51]. In the soluble fraction, only the whey proteins α-lactalbumin and β-lactoglobulin remain.

In the soy samples (Figure 9c), the top three bands might correspond to subunits of the trimeric 7S globulin, also called β-conglycinin: α-globulin at around 86 kDa, α’-globulin at ca. 77 kDa, and β-globulin around 57 kDa. The bands around 33 and 32 kDa might correspond to the acidic and the band around 27 kDa to the basic subunit of the 11S globulin glycinin. Glycinin is a hexamer consisting of five different kinds of subunits, each of which comprises an acidic and a basic subunit. In the soluble fraction, β-amylase at ~61 kDa, lectin at ~33 kDa, and Kunitz trypsin inhibitors at ~20 kDa can be seen [52].

## 4. Discussion

Soy- and coconut-based yoghurt alternatives currently dominate the market, but products from alternative sources are on the rise [17,53]. It has been shown in this study that lentil protein isolate is a well-suited base material for a plant-based yoghurt alternative with a low environmental impact. Compared to the fermentation of cow’s milk, acidification of the lentil emulsion and the soy control was slightly slower, and the present sucrose was not entirely metabolised. Even though similar pH values could be reached, the TTA values were significantly lower. As a LAB culture for traditional cow’s milk yoghurt was used for fermentation, and LAB have complex nutritional requirements [54], these results are not surprising, however. Textural analysis showed higher values for firmness and consistency for the fermented lentil samples than dairy. In contrast, the cohesiveness and viscosity of the lentil samples were not different from the dairy control. Firmness is the force necessary to break the protein gel, while consistency expresses the thickness of the gel [55]. Cohesiveness and viscosity are measured when the probe disc is pulled out of the sample. The resistance of the sample to flow off of the disc indicated viscosity, the maximum negative force necessary to pull up the probe shows the cohesiveness of the sample. The viscosity index is calculated from both viscosity and cohesiveness, and the higher the value, the more resistant the sample is against the extraction of the probe [26,55]. Fermented lentil emulsions, therefore, produce a stiffer and thicker gel than dairy, while flow behaviour is comparable to that of a dairy product. The three-interval thixotropy test showed that the fermented lentil gel, as well as the soy control, were less sensible to shear stress than the dairy control. The faster gel formation of the lentil emulsion during oscillatory analysis compared to dairy can be explained by lentil protein’s steep decrease in solubility at a pH below 7, and minimum solubility is already reached at pH 6 [33]. Soy protein solubility is still high at pH 6, and a minimum is only reached around pH 4 [56]. And even though the dairy sample had slightly faster acidification than the other samples (see Figure 1), gel-formation was slower. This can be explained by the gelation mechanism of caseins, which include the solubilisation of colloidal calcium phosphate, as well as a decreased negative charge of casein micelles below pH 6, both of which weakens and ultimately dissociates the casein micelle structure. Finally, only at a pH below 5, the dissociated casein molecules form a gel network, due to hydrophobic and electrostatic interactions [35]. CLSM provided insight into the microstructure of the protein network after fermentation. The lentil protein gel matrix was slightly denser with smaller pores than the dairy control, whereas the softer soy control showed a very loose gel structure with large pores. As small pore size indicates higher protein network strength [57], the CLSM results confirm the results of textural and rheological analyses. While lentils and other pulses usually contain large levels of FODMAPs and their consumption should, therefore, be limited by individuals with gastrointestinal disorders, such as IBS [58], a large proportion of these FODMAPs is removed during the process of the isoelectric precipitation isolation of the proteins from the whole lentil flour, as FODMAPs are water soluble [44]. The cut-off value for the amount of GOS per serving that does not cause gastrointestinal symptoms in IBS patients has been established below 200 mg [59]. A typical serving of 150 g of lentil yoghurt alternative contains only 79.5 mg of GOS, which is far below the cut-off value, while the soy yoghurt alternative contains 573 mg of GOS per serving, thus far exceeding the cut-off value. The total polyol cut-off value is 400 mg, and that of EF is 150 mg per serving [59]; the lentil product, as well as the soy control, fall well below both cut-off levels. The dairy yoghurt exceeds the lactose cut-off value of 1000 mg per serving more than five-fold. In summary, neither the fermented dairy nor the soy controls are advisable for IBS patients because of their high FODMAP content, while FODMAP content in a serving of the lentil yoghurt is well below the acceptable FODMAP threshold. Fermentation did not cause any significant further reduction in FODMAP content. It has been shown that proteolytic activity in lactic acid fermentation of pulse proteins can cause protein degradation, altering the protein digestibility by increasing protein solubility and the number of free amino acids [60,61]. Lab-on-a-Chip capillary electrophoresis indicated that the LAB used in this study did not degrade the lentil protein, so no significant changes in nutritional protein quality by fermentation are expected in this case. Due to water-soluble pigments from the lentil seed coat, such as anthocyanins and flavonoids [46], lentil protein isolate emulsion is of a pale pink colour, but a high whiteness index is a desirable trait in plant-based dairy alternatives. With fermentation, the whiteness index of the fermented lentil samples could be increased to a level higher than that of the soy control. With the drainage method, the lentil samples showed low liquid expulsion, but higher liquid expulsion values with the centrifugation method. The drainage method represents liquid drainage when a large surface area is available and is especially relevant for producing strained yoghurt products, such as Greek yoghurt. However, the centrifugation test, especially when performed at relatively low centrifugation speed, is a better representation of spontaneous liquid separation. Spontaneous liquid separation is caused by gel contraction during the gelling process [35,62]. Therefore, future research into lentil protein applications in dairy alternatives where high gel strength is desirable, such as sour cream or cream cheese alternatives [63,64], is of interest.

## 5. Conclusions

Demand for dairy alternatives is increasing, and the variety of plant-based yoghurt alternatives is growing. The potential of lentil protein for the development of dairy yoghurt alternatives is promising. The acidification of a lentil protein isolate emulsion by LAB fermentation resulted in a very firm gel formation. Textural and rheological analyses showed that a structure comparable to that of dairy yoghurt was achieved. Due to low FODMAP levels and a protein content equal to that of dairy milk, lentil protein isolate is suitable as a base ingredient to produce a nutritious alternative to yoghurt. Due to its gelling properties, lentil protein isolate revealed a high potential for future application in other fermented dairy alternative products. However, more research is needed to identify LAB with high fermentation performance for the lentil protein substrate. 

## Figures and Tables

**Table 1:** Lactic acid content, sucrose content as measured by HPLC, and microbial cell count displayed as colony forming units (CFU) in non-fermented and fermented samples and controls. N.d.: not detected or below 0.6 mg/100 g for lactic acid and below 0.5 mg/100 g for sucrose. Values are displayed as means plus minus standard deviation. Values within the non-fermented and fermented groups with different superscript letters (capital letters for non-fermented, lowercase letters for the fermented group) are significantly different (*p* ≤ 0.05).

		Lactic Acid			Sucrose			CFU/mL		
		[mg/100 g]			[mg/100 g]					
Non-fermented	Lentil	n.d.			780	±	32 ^A^	1.36 × 10^7^	±	5.64 × 10^6 A^
	Dairy	n.d.			748	±	144 ^A^	2.65 × 10^7^	±	1.61 × 10^6 A^
	Soy	n.d.			704	±	8 ^A^	2.12 × 10^7^	±	1.45 × 10^7 A^
Fermented	Lentil	244	±	8 ^a^	483	±	24 ^a^	3.54 × 10^7^	±	2.98 × 10^7 a^
	Dairy	603	±	16 ^b^	n.d.			7.09 × 10^8^	±	6.50 × 10^8 a^
	Soy	339	±	9 ^c^	328	±	5 ^b^	3.02 × 10^7^	±	2.98 × 10^7 a^

**Table 2:** Results of the thixotropy test (ΔG′ [%] and ΔG″ [%]) and the frequency sweep (dlog G′/dlog ω, dlog G″/dlog ω, dlog|η*|/dlogω). Values within columns with different superscript letters are significantly different (*p* ≤ 0.05).

	ΔG′ [%]			ΔG″ [%]			Dlog G′/Dlog ω			Dlog G″/Dlog ω			Dlog|η*|/Dlogω		
Lentil	55.30	±	3.57 ^a^	54.97	±	1.95 ^a^	0.147	±	0.003 ^a^	0.208	±	0.007 ^ab^	−0.850	±	0.003 ^a^
Dairy	69.63	±	3.48 ^b^	67.84	±	3.63 ^b^	0.164	±	0.019 ^a^	0.204	±	0.009 ^a^	−0.831	±	0.018 ^a^
Soy	53.73	±	0.63 ^a^	51.44	±	0.56 ^a^	0.113	±	0.003 ^b^	0.221	±	0.003 ^b^	−0.882	±	0.003 ^b^

**Table 3:** Colour of the fermented samples, using the CIE L*a*b* colour space, with L* representing lightness (0 = black, 100 = white); a* showing green (−500) to red (+500); b* ranging from blue (−200) to yellow (+200); and whiteness index (WI). Values within a column of the fermented (lowercase letters) or unfermented product (capital letters) with different superscript letters are significantly different (*p* ≤ 0.05).

		L*			a*			b*			WI		
Non-fermented	Lentil	81.21	±	0.24 ^A^	1.36	±	0.22 ^A^	6.30	±	0.17 ^A^	80.14	±	0.23 ^A^
	Dairy	93.83	±	0.02 ^B^	−4.70	±	0.64 ^B^	6.93	±	0.01 ^B^	89.59	±	0.30 ^B^
	Soy	88.74	±	0.19 ^C^	1.61	±	0.11 ^C^	13.44	±	0.02 ^C^	82.40	±	0.15 ^A^
Fermented	Lentil	87.90	±	0.24 ^a^	1.65	±	0.28 ^a^	7.57	±	0.28 ^a^	85.63	±	0.25 ^a^
	Dairy	96.20	±	0.10 ^b^	−3.79	±	0.10 ^b^	9.35	±	0.43 ^b^	89.22	±	0.37 ^b^
	Soy	92.88	±	0.03 ^c^	−0.77	±	0.07 ^c^	13.26	±	0.20 ^c^	84.93	±	0.17 ^c^

**Table 4:** FODMAP content as quantified by HPAEC-PAD. All results are given as means in mg/100 g sample ± standard deviations. N.d.: not detected or below 5 mg/100 g. Values within a column of the fermented (lowercase letters) or unfermented product (capital letters) with different superscript letters are significantly different (*p* ≤ 0.05).

		Lactose	Glucose	Fructose	EF
Non-fermented	Lentil	n.d.	6 ± 1 ^A^	n.d.	-
	Dairy	5257 ± 148	n.d.	n.d.	-
	Soy	n.d.	7 ± 0 ^A^	n.d.	-
Fermented	Lentil	n.d.	19 ± 4 ^ab^	26 ± 7 ^ab^	8 ± 4 ^a^
	Dairy	4807 ± 23	23 ± 10 ^a^	40 ± 10 ^a^	17 ± 20 ^a^
	Soy	n.d.	10 ± 0 ^b^	15 ± 1 ^b^	5 ± 1 ^a^
	**Polyols**				
		**Xylitol**	**Sorbitol**	**Mannitol**	**Ʃ** **Polyols**
Non-fermented	Lentil	9 ± 0	22 ± 10 ^A^	n.d.	30 ± 11 ^A^
	Dairy	n.d.	n.d.	n.d.	n.d.
	Soy	n.d.	7 ± 2 ^B^	n.d.	11 ± 2 ^B^
Fermented	Lentil	n.d.	n.d.	n.d.	n.d.
	Dairy	n.d.	n.d.	n.d.	n.d.
	Soy	n.d.	9 ± 0	n.d.	13 ± 0
	**GOS**				
		**Raffinose**	**Stachyose**		
Non-fermented	Lentil	n.d.	62 ± 2 ^A^		
	Dairy	n.d.	n.d.		
	Soy	102 ± 2	310 ± 8 ^B^		
Fermented	Lentil	n.d.	53 ± 2 ^a^		
	Dairy	n.d.	n.d.		
	Soy	89 ± 28	293 ± 2 ^b^		
